# Computed Tomography-Based Radiomics Using Tumor and Vessel Features to Assess Resectability in Cancer of the Pancreatic Head

**DOI:** 10.3390/diagnostics13203198

**Published:** 2023-10-13

**Authors:** Geke Litjens, Joris P. E. A. Broekmans, Tim Boers, Marco Caballo, Maud H. F. van den Hurk, Dilek Ozdemir, Caroline J. van Schaik, Markus H. A. Janse, Erwin J. M. van Geenen, Cees J. H. M. van Laarhoven, Mathias Prokop, Peter H. N. de With, Fons van der Sommen, John J. Hermans

**Affiliations:** 1 Department of Medical Imaging, Radboud Institute for Health Sciences, Radboud University Medical Center, 6525 GA Nijmegen, The Netherlands; 2 Department of Electrical Engineering, Eindhoven University of Technology, 5612 AZ Eindhoven, The Netherlands; 3 Department of Plastic and Reconstructive Surgery, Saint Vincent’s University Hospital, D04 T6F4 Dublin, Ireland; 4 Image Sciences Institute, University Medical Center Utrecht, 3584 CX Utrecht, The Netherlands; 5 Department of Gastroenterology and Hepatology, Radboud Institute for Molecular Life Sciences, Radboud University Medical Center, 6525 GA Nijmegen, The Netherlands; 6 Department of Surgery, Radboud Institute for Health Sciences, Radboud University Medical Center, 6525 GA Nijmegen, The Netherlands

**Keywords:** pancreas, adenocarcinoma, computed tomography, radiomics, resectability, oncology

## Abstract

The preoperative prediction of resectability pancreatic ductal adenocarcinoma (PDAC) is challenging. This retrospective single-center study examined tumor and vessel radiomics to predict the resectability of PDAC in chemo-naïve patients. The tumor and adjacent arteries and veins were segmented in the portal-venous phase of contrast-enhanced CT scans, and radiomic features were extracted. Features were selected via stability and collinearity testing, and least absolute shrinkage and selection operator application (LASSO). Three models, using tumor features, vessel features, and a combination of both, were trained with the training set (*N* = 86) to predict resectability. The results were validated with the test set (*N* = 15) and compared to the multidisciplinary team’s (MDT) performance. The vessel-features-only model performed best, with an AUC of 0.92 and sensitivity and specificity of 97% and 73%, respectively. Test set validation showed a sensitivity and specificity of 100% and 88%, respectively. The combined model was as good as the vessel model (AUC = 0.91), whereas the tumor model showed poor performance (AUC = 0.76). The MDT’s prediction reached a sensitivity and specificity of 97% and 84% for the training set and 88% and 100% for the test set, respectively. Our clinician-independent vessel-based radiomics model can aid in predicting resectability and shows performance comparable to that of the MDT. With these encouraging results, improved, automated, and generalizable models can be developed that reduce workload and can be applied in non-expert hospitals.

## 1. Introduction

Pancreatic ductal adenocarcinoma (PDAC) is the fourth leading cause of cancer-related death, with the lowest 5-year overall survival (OS) rate of approximately 12%, which has only slowly improved in the past 40 years [1]. Surgical resection is the only potentially curative treatment, but most patients are deemed ineligible for resection due to metastases and/or locally advanced pancreatic cancer (LAPC) [2]. Of the patients undergoing surgery with curative intent, 18–35% are considered irresectable during surgery due to unexpected metastases or vessel invasion [3,4]. Accurate assessment of vessel invasion is crucial to determining resectability and preventing futile surgery.

In current clinical practice, the staging of PDAC is based on the radiologist’s assessment of vessel involvement via contrast-enhanced computed tomography (CECT). Several guidelines predict resectability, such as the National Comprehensive Cancer Network, the New Japanese Classification of Pancreatic Cancer, and the Dutch Pancreatic Cancer Group (DPCG) [5,6,7]. All three guidelines define three groups, (1) resectable, (2) borderline resectable, and (3) irresectable (i.e., LAPC), based on extent of tumor–vessel contact. The cut-off values for the contact degrees for each group vary between guidelines, and the classification of resectability is subject to interobserver variability [8]. Moreover, subjective terms like ‘reconstructable’ are used, which can differ between institutions and surgeons. A tool to assess resectability that is more reproducible and observer-independent may therefore be helpful.

Radiomics is a process wherein medical images are converted into minable high-dimensional data that can be used for quantitative image analysis [9]. With radiomics, additional information about the tumor and its surroundings can be captured compared to visual analysis alone, which is inherently more subjective. 

Quantitative CECT analysis of PDAC has shown that texture features are associated with OS in both resected and non-resected patients [10,11]. Additionally, studies have demonstrated that radiomics can improve the prediction of the resection margin (R0 or R1) [12,13]. Although the assessment of vessel involvement by the radiologist is the current method to assess resectability in PDAC, there is limited literature on the use of segmentations of adjacent vessels of PDAC for radiomics. One study included segmentations of the superior mesenteric artery and perivascular tissue [14], and two studies included venous segmentations [12,15]. Furthermore, the DeepPrognosis project proposed a deep learning multi-task convolutional neural network framework including tumor and vessel information on automated segmentations to predict OS and the resection margin [16]. However, in this project, segmentations were not validated by an expert radiologist, and they used the simple representation of tumor–vessel relationship (number of contacted voxels). 

In this study, we used two types of features, (1) tumor features and (2) handcrafted vessel features, to quantify the tumor–vessel contact of adjacent arteries and veins. To our knowledge, this is the first radiomics study to include both the 3D tumor and vessel features of all relevant veins and arteries to predict the resectability of PDAC. We hypothesize that a radiomics model with both tumor and vessel features will accurately predict resectability in chemo-naïve patients with PDAC of the pancreatic head.

## 2. Materials and Methods

**Figure 1:**
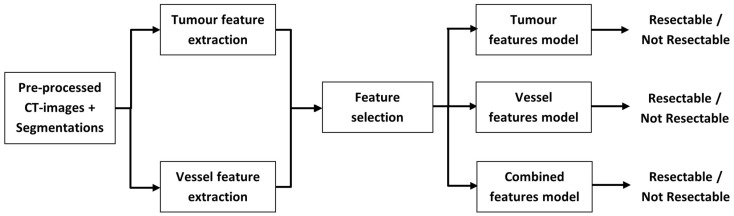
Study workflow to develop the three models for resectability prediction.

This retrospective single-center study consisted of four stages, illustrated in Figure 1. First, the tumor and surrounding vessels were manually segmented on the portal-venous phase of CECTs, which were pre-processed to harmonize voxel size. Second, tumor and vessel features were extracted. Third, feature selection was performed. Finally, utilizing the selected features, three prediction models (a tumor features model, a vessel features model, and a combined features model) were trained and validated with a separate, unseen test set. The results were compared to those of the multidisciplinary team (MDT). Additionally, all these steps were repeated on a subset of resected tumors to develop prediction models for the surgical resection margin. 

### 2.1. Study Population

This retrospective study consecutively included patients diagnosed between January 2015 and June 2018 with proven PDAC of the pancreatic head (cytology or histology). The majority of patients were referred to our tertiary pancreatic cancer center, and all patients were chemo-naïve. We excluded patients for whom surgery with curative intent was not possible due to metastases or a poor performance status, patients with a stent or endoprosthesis in the pancreatic or common bile duct present on CECT, patients to whom neoadjuvant treatment had been administered, and patients for whom the interval between CECT and surgery (if applicable) was more than eight weeks. We validated the model results in a test set of 15 patients diagnosed between June 2018 and February 2021 who met the same criteria. To obtain a balanced test set, cases were selected in the same ratio as the training set with regard to MDT classification and whether or not surgery was performed.

### 2.2. Clinical Information

Demographic data (age and sex), resectability by MDT according to DPCG guidelines [6], and CT-reported tumor size were collected. The DPCG guidelines are as follows: resectable: no arterial contact and venous contact ≤90°; borderline resectable: arterial contact ≤90° and/or venous contact 90–270° and no occlusion; irresectable: arterial contact >90° and/or venous contact >270° or occlusion. The arteries assessed for these guidelines were the superior mesenteric artery (SMA), celiac axis, and common hepatic artery (CHA); the veins were the superior mesenteric vein (SMV) and portal vein (PV). Surgical records and MDT reports served as the reference standard for resectability. Patients who underwent surgery with curative intent but were deemed irresectable during surgery due to unexpected vascular invasion were classified as irresectable. Additionally, patients who did not undergo surgery because they were deemed irresectable by clinical consensus from the MDT were also classified as irresectable. 

### 2.3. Image Acquisition and Segmentation

**Figure 2:**
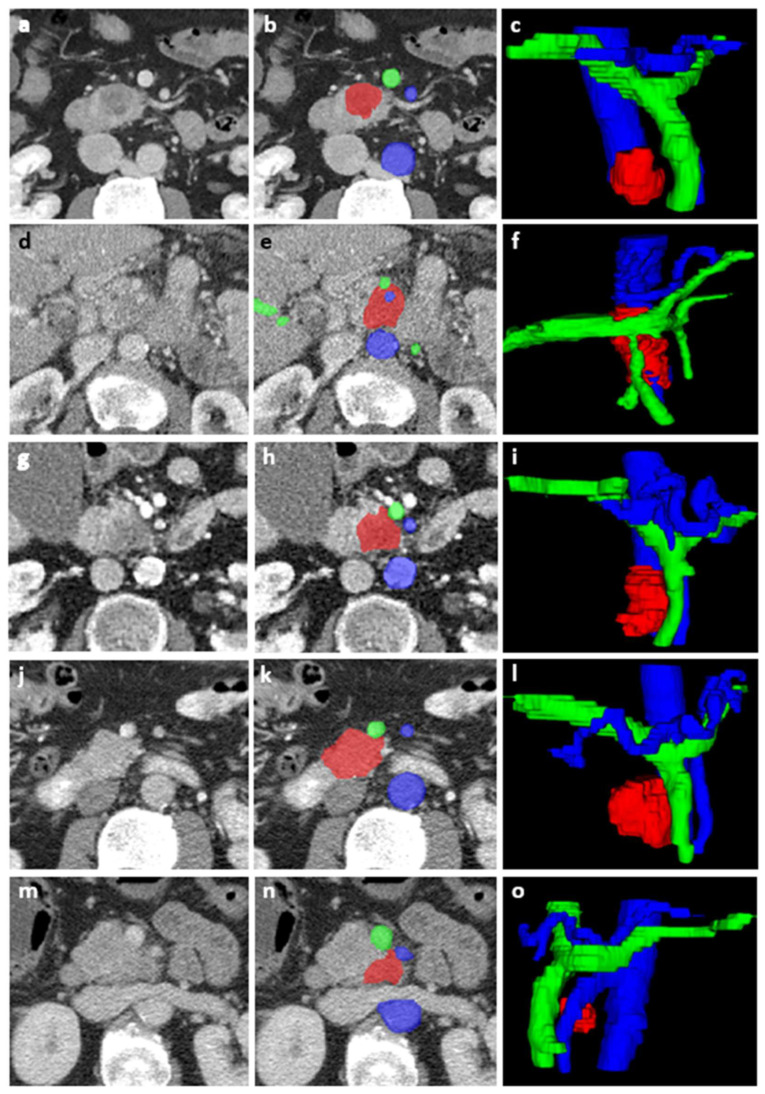
Segmentations of five example cases included in this study. For each case, an axial CECT slice (with and without segmentations) and a 3D view of the segmentation are shown (tumor in red, arteries in blue, and veins in green). First (**a**–**c**), a true positive case, without any vessel contact, where the three models and the MDT correctly predicted resectability. Second (**d**–**f**), a true negative case, with both arterial and venous tumor–vessel contact, where the three models and the MDT correctly predicted irresectability. Third (**g**–**i**), a false positive case, with venous tumor–vessel contact, where the three models and the MDT predicted resectability, but the tumor was irresectable during surgery due to encasement of the VMS. Fourth (**j**–**l**), a false negative case, with venous tumor–vessel contact, where the three models predicted irresectability, the MDT predicted resectability, and the tumor was resected. Finally, (**m**–**o**), a true negative case, with venous and arterial tumor–vessel contact, where the three models predicted irresectability and the MDT predicted resectability, but the tumor was irresectable during surgery due to the encasement of VMS and AMS.

We used multi-centric imaging data for our analysis. Pre-operative imaging was conducted at 11 institutions with varying CT scan protocols, depending on the patients’ presenting symptoms. Contrast volume, timing, and injection rate, as well as slice thickness, varied across the dataset. However, a portal-venous-phase CECT was available for all patients. This phase was used for segmentation and further analysis. Trained medical students (M.H., D.O., C.S.), supervised by a PhD student (G.L.), performed the segmentation and labeling of the tumor and adjacent arteries and veins using ITK-SNAP (version 3.8.0, www.itksnap.org) [17]. Segmentations were performed on multiple consecutive slices, including the entire tumor, and vessels well beyond the tumor, to obtain a 3D segmentation for analysis. All segmentations were manually verified by a radiologist with 23 years of experience in abdominal radiology (J.H.). Examples of the segmentations are shown in Figure 2. Segmentations were performed blinded for clinical data. To differentiate the tumor from healthy pancreatic tissue, differences in contrast-enhancement, and signs such as a dilated pancreatic or common bile duct, were used. For isoattenuating tumors with unclear boundaries, if available, additional contrast phases or MRI-imaging were reviewed to define tumor boundaries. The segmented veins were the PV, SMV, and splenic vein (SV). The segmented arteries were the abdominal aorta, celiac axis, CHA, SMA, and splenic artery (SA). Images and segmentations were rescaled to encompass the resolution differences of the varying scanning protocols. To account for pixel spacing differences, linear interpolation for CT-images and nearest neighbor interpolation for segmentations were used, resulting in isotropic voxels of 1 mm^3^. 

### 2.4. Tumor Features 

Tumor feature extraction was performed on the tumor segmentations using Python’s PyRadiomics package (version 3.0.1) [18]. The 107 extracted features can be divided into three groups: 18 first-order intensity features, 14 shape-based features (2D and 3D), and 75 texture features. Table S1 in the supplementary files provides a complete list of extracted features. Feature stability, with respect to tumor segmentation, was assessed through an interobserver study. Three radiologists, with, respectively two, three, and 23 years of experience, separately segmented the tumor on nine randomly chosen scans from the database, blinded to clinical data. Their average DICE score for tumor segmentation was 0.71 (range 0.5–09). The intraclass correlation coefficient (ICC) of all tumor features was calculated according to McGraw and Wong [19]. Features with an ICC < 0.75 were considered unstable and were excluded [12,20]. The remaining features were standardized using z-score normalization and were investigated for collinearity. If the Spearman correlation coefficient between two features was over 0.9, one feature was randomly removed due to high collinearity. 

### 2.5. Vessel Features 

**Figure 3:**
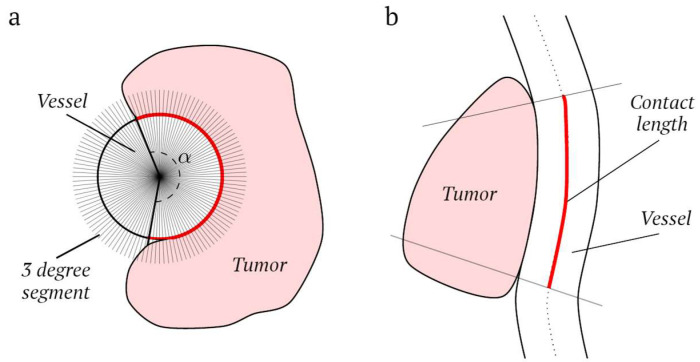
A schematic representation of the method used to extract (**a**) the maximum angle (α) of encasement, determined with 3-degree segments, and (**b**) the maximum tumor–vessel contact length.

To quantify tumor–vessel contact, we developed a tool that extracts additional vessel features using neighboring tumor and vessel voxels. The extracted vessel features included the maximum angle of encasement, maximum tumor–vessel contact length, and tumor–vessel contact area. These vessel features quantify all aspects of tumor–vessel contact. The maximum angle of encasement was extracted from the plane perpendicular to the longitudinal vessel axis determined from the central lumen line. Subsequently, each perpendicular plane was divided into segments of 3 degrees each, and the plane with tumor voxels in most of these segments was considered the plane with the maximal angle of encasement (Figure 3a). The maximum tumor–vessel contact length was determined based on the maximal number of consecutive voxels with tumor–vessel contact (Figure 3b). Voxels less than 5 mm apart in the direction of the central lumen line were considered consecutive. To determine the tumor–vessel contact area, all vessel voxels directly connected to a tumor voxel were extracted. Multiple contact areas may exist. The tumor–vessel contact area is the sum of all contact areas. All vessel features were separately extracted for the arteries and veins, leading to six vessel features in total (three arterial and three venous). Feature selection on these six features was performed by analyzing collinearity, similar to the tumor features, as shown in Table S3 in the supplementary files.

### 2.6. Model Development

The least absolute shrinkage and selection operator (LASSO) [21] was used on the training set for additional feature selection, leading to three different feature sets: (1) tumor features, (2) vessel features, and (3) a combination of tumor and vessel features. The LASSO hyperparameter lambda was optimized using five-fold cross-validation. For each feature set, a support vector machine (SVM) classifier was trained on the full training set, using a linear kernel to keep the model complexity low. The box constraint hyperparameter was optimized using Bayesian optimization, with the MATLAB (The MathWorks Inc. version 9.10 (R2021a), Natick, Massachusetts, United States of America) classification learner toolbox. Receiver operating characteristic (ROC) curves with the area under the curve (AUC) were created for the three models. The ROC curves of the three models were compared using the method established by Hanley and McNeil [22]. The performance of the three SVM classifier models for the training set was determined by calculating the sensitivity, specificity, positive predictive value (PPV), and negative predictive value (NPV) at the optimal point on the ROC curves according to the Youden index [23]. The results were validated by calculating the performance for the test set using crosstabs. The performance of the MDT was also calculated using crosstabs. Resectable and borderline resectable were combined to facilitate sensitivity and specificity analysis. The statistical significance of the differences in sensitivity and specificity between the models and the MDT were evaluated using the McNemar test. 

### 2.7. Subset Analyses: Resection Margin Status

For a subset of only resected patients, the entire study workflow of feature selection (collinearity analysis and LASSO) and the training of three SVM models were repeated to predict resection margin status (R0 or R1/2). The histopathological resection margin status was used as the ground truth. The sensitivity, specificity, positive predictive value (PPV), and negative predictive value (NPV) for the training set were calculated at the optimal point from the produced receiver operating characteristic (ROC) curves using the Youden index. These results were also validated using the resected patients of the test set. 

The histopathological resection margin status (R0, R1, R2) for this subset analysis was microscopically defined according to the UK Royal College of Pathologists: R0 if there was margin clearance of >1 mm, except for the anterior pancreatic surface, for which 0 mm clearance was sufficient (since this is an anatomical surface, not a surgical margin); R1 if the margin was ≤1 mm; and R2 if there was macroscopic tumor residue during surgery [24]. In the subset analysis, R1 and R2 were combined to create a binary outcome.

## 3. Results

### 3.1. Patients

**Figure 4:**
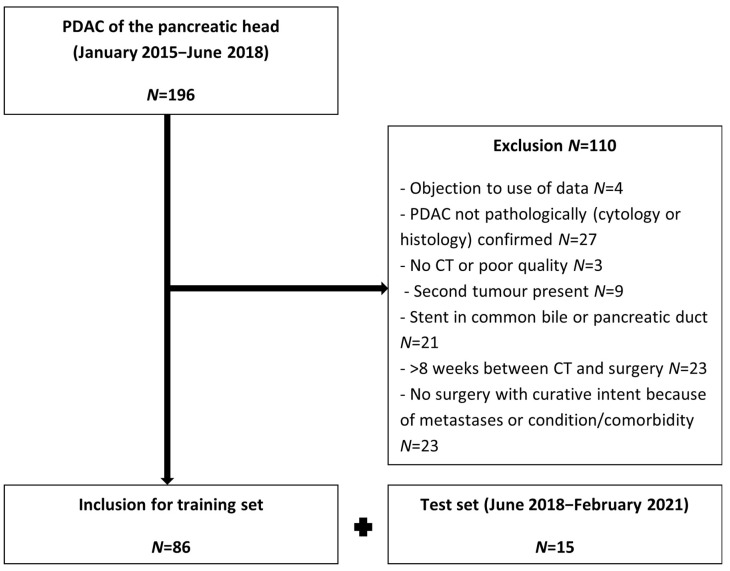
Flowchart of patient inclusion and exclusion.

Figure 4 shows a flowchart of the patient selection and inclusion. The training set included 86 patients, and the test set included 15. Table 1 displays the patient and tumor characteristics; there were no significant differences between the training and test set. Forty-two (48%) patients in the training set and eight (53%) from the test set underwent surgery with curative intent, of which, respectively, 31/42 (74%) and 7/8 (88%) were resected and 11/42 (26%) and 1/8 (13%) were deemed irresectable during surgery due to advanced vascular invasion. The other 46 (52%) patients in the training set and seven (47%) in the test set did not undergo surgery as their tumors were deemed irresectable by the MDT.

### 3.2. Feature Extraction and Selection

**Figure 5:**
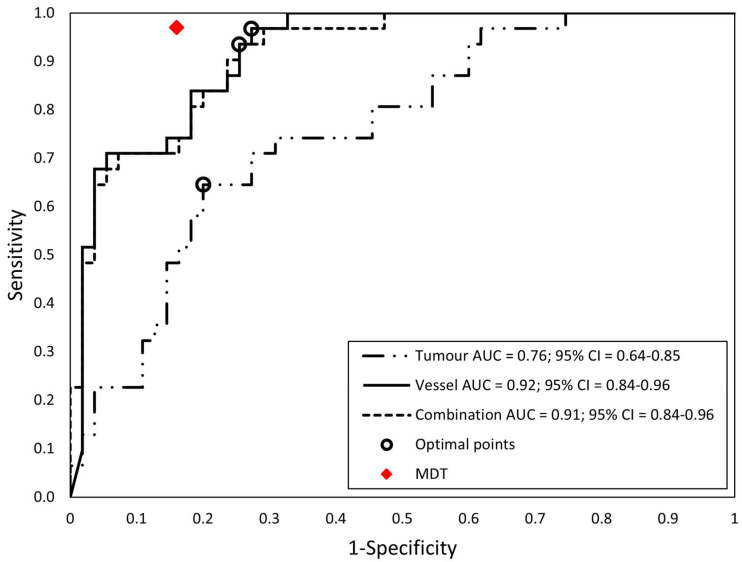
The ROC curves of the three SVM models to predict resectability (training set, *N* = 86). The optimal point for each classifier according to the Youden index is indicated with a circle.

A total of 88 of 107 tumor features had high stability (ICC > 0.75). After collinearity assessment, 29 tumor features remained. Three of six vessel features showed high collinearity. The three remaining vessel features were the venous angle of encasement, venous tumor–vessel contact length, and arterial angle of encasement. The Supplementary files show details of stability and collinearity analysis of the tumor and vessel features (Tables S1–S3, and a separate Excel matrix file in Supplementary files). LASSO was used on the remaining 29 tumor and three vessel features to select the three feature sets. The tumor features model consists of six features, the vessel features model of three features, and the combined features model of four features (one tumor and three vessel features). In both the vessel features model and the combined features model, two of the three vessel features were venous features. The included features for each model are shown in Table 2. Figure 5 shows the ROC curves with optimal points of the training set. Table 3 displays the performance of the models at the optimal point, the performance of the MDT, and the comparison between the models and the MDT. The areas under the curve (AUCs) of the vessel features model and the combined features model were significantly better than that of the tumor features model (*p* < 0.01 and *p* = 0.02, respectively), and there was no significant difference between the AUCs of the vessel features model and the combined features model (*p* = 0.82). Table 3 also displays the validation of the models with the test set. Figure 2 shows five example cases.

### 3.3. Subset Analyses: Resection Margin Status

To develop the models for the prediction of resection margin status, 38 resected patients were included, including 31 from the training set and 7 from the test set. In the training set, 14 (45%) patients had an R0 margin, 16 (52%) patients an R1 margin, and 1 (3%) patient had an R2 margin. In the test set, 5 (71%) patients had an R0 margin, 2 (29%) patients an R1 margin, and none an R2 margin.

**Figure 6:**
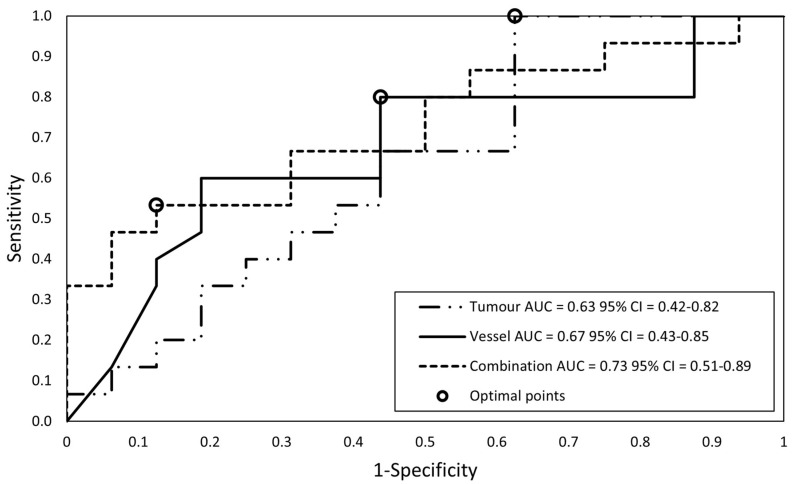
The ROC curves of the three SVM classifiers to predict resection margin status (R0 vs. R1/2; training set, N = 31). The optimal point for each classifier according to the Youden index is indicated with a circle.

After feature selection including the LASSO method again, three models were created: the tumor feature model consists of four features, the vessel feature model consists of two features, and the combined features model consists of five features (four tumor, one vessel). Figure 6 shows the ROC curves with optimal points of the training set. The performance of the models at the optimal points (sensitivity, specificity, PPV, and NPV) is displayed in Table 4. There was no significant difference between the AUCs of the three models. The results of the three trained SVM classifiers were validated using the seven resected patients of the test set; these results are also displayed in Table 4.

## 4. Discussion

In this retrospective study, we created three different radiomics models using tumor features, vessel features, and a combination of both that predicted resectability with an AUC of 0.76, 0.92, and 0.91, respectively, for a training set. The vessel features and combined features models showed the best performance, with a sensitivity of 97% and 94%, specificity of 73% and 75%, PPV of 67% and 67%, and NPV of 98% and 95% for the training set, respectively. These models were significantly better than the tumor features model. Validation with the test set led to a sensitivity of 100% and specificity of 88% for both models. The models showed a high NPV, which is useful for preventing futile surgery. The results of the vessel features and combined features models were comparable to the results of the MDT of our tertiary expertise center.

Studies show that there is considerable interobserver variability between radiologists and MDTs when determining resectability [8,25]. Two meta-analyses analyzed radiologists’ prediction of resectability (without radiomics). They found a pooled sensitivity of 63% and 77% and specificity of 81% and 92% for diagnosing vessel invasion with CT [26,27]. The results of our two best models to predict resectability reached a higher sensitivity (94–97%) but lower specificity (73–75%). The benefits of a radiomics model are that it is clinician-independent, and does not rely on locally available expertise. Our model can be re-trained with data from any center to adapt it to local conditions, and their predictions could be used to support the MDTs decision.

To our knowledge, this is the first radiomics study to combine the tumor and vessel features of all relevant vessels to predict resectability. Three recent studies used radiomics with limited vessel information to predict resection margins. The first used 2D segmentations of PV margins on three slices per patient to predict R0 or R1, and they reached an AUC of 0.86, sensitivity of 76%, and specificity of 90% [13]. The second used 2D tumor segmentations on a slice with maximum tumor–vein contact to predict vein invasion, and they reached an AUC of 0.85, sensitivity of 79%, and specificity of 74% [15]. The third used 3D segmentations of the tumor, SMA, and perivascular tissue surrounding the SMA to predict the SMA resection margin, and they reached an AUC of 0.71, sensitivity of 62%, and specificity of 77% [14]. The first two studies analyzed tumor–vein margins and the third study analyzed arterial margins (the SMA). In our study, we included both veins and arteries. After feature selection, two venous features (contact length and angle) and only one arterial feature (contact angle) were included in our models. Furthermore, these three studies used resection margins as the outcome, while we used resectability as our primary outcome. We performed an additional subset analysis on the resected patients to develop models to predict the histopathological resection margin. In this analysis, the model with a combination of tumor and vessel features performed best, with an AUC of 0.73, and for the training set, a sensitivity of 53% and specificity of 88%. These results should be interpreted with care because of the small subset available for this analysis, but they show that there might be more potential in our models than the prediction of surgical resectability alone.

In clinical practice, surgical exploration is not performed in patients deemed irresectable by the MDT. In our study, 52% of patients did not undergo surgery with curative intent due to clinical consensus by the MDT that a tumor was irresectable. This causes bias in favor of the MDT’s performance with probable overestimation of the MDT’s sensitivity, caused by an underrepresentation of false negative cases (MDT classification irresectable, but surgically resectable). In four cases surgery was not performed because the tumor was deemed irresectable by the MDT, but the model classified the tumor as resectable. These predictions of the model were classified as false positives, but the true resectability is unknown because surgery was not attempted.

Currently, strategies are shifting towards neoadjuvant treatment, which can increase R0 resections and significantly improve OS [28,29]. Determining resectability after neoadjuvant treatment is challenging, as the differentiation of benign treatment-related changes (pancreatitis or fibrosis) and residual tumor is difficult, and RECIST criteria are not reliable for assessing treatment response [29,30,31,32]. One study showed that an increased tumor attenuation on CT after FOLFIRINOX might be a predictor for R0 resection [33]. Although in our study, vessel features proved more useful, the model with tumor features only reached a sensitivity of 65% and specificity of 80%. This shows that there is also relevant information within the tumor itself, without specific vessel information. Tumor features could therefore potentially be used for the evaluation of treatment response, as shown in two other studies on CT-based radiomics. The first found promising results using tumor texture analyses before and after neoadjuvant treatment to predict resectability and prognosis in PDAC [34]. The second used radiomics to predict resectability for LAPC after chemotherapy and radiotherapy with an AUC of 0.94 [35]. Our approach of combining tumor and vessel features has potential for other applications, such as detecting changes that indicate treatment response.

Our exploratory study has several limitations. First, the variety of CT scanners and scanning protocols from multiple institutions could have affected image noise and texture, and therefore the feature analysis [36,37]. On the other hand, our results show that good model performance is possible even when different scanners and protocols are included, which also reflects daily clinical practice. Nevertheless, future research methods should account for inter-scanner and -protocol variability, as proposed by the ComBat harmonization strategy [38]. Second, we only used the portal-venous phase because it was available for all patients. In the parenchymal phase, the arterial wall can be delineated better; therefore, including this phase could improve results. The DeepPrognosis project shows that a deep learning model with three phases (non-contrast, pancreatic, and venous phase) performs better than a single-phase model; this is probably also true for radiomics models [16]. Third, we did not perform external validation. We validated the results with a test set, but these were cases included from the same institution. Fourth, we focused on resectability, not on the resection margin, which was only analyzed in a smaller subset. We acknowledge that resectability is not only variably defined in different guidelines, but also depends on patient condition and preference of surgical approach in each expert center. However, for the resection margin, which is used as an outcome in most other studies, various meanings are also used that lead to interobserver variability and differences in patient management. The R status is often interpreted as an indication of surgical quality, but studies have shown that the quality of the pathological examination determines the R1 rate, not the surgical quality [39]. Furthermore, resection is a treatment with curative intent and a clear improvement of prognosis, while no resection leads to palliative treatment with a worse prognosis. This makes surgical resectability a good and clear surrogate marker for outcomes. Finally, all CT scans were manually segmented, which is very time-consuming and carries potential interobserver effects. To minimize these effects in the current study, one experienced radiologist checked all segmentations. Additionally, feature robustness was analyzed via an interobserver study. To reduce segmentation time, interobserver variability, and segmentation bias, a (semi)automatic segmentation tool should be developed for future research. Our research team made promising first steps towards such a tool [40].

A strength of our study is that we used multiple slice segmentations of the entire tumor and all adjacent vessels, which allowed us to perform 3D analysis, unlike several other studies using segmentations of the tumor only on a single axial slice (2D analysis), for texture analysis in PDAC [41,42,43,44]. A study showed that most texture features differ significantly when comparing 2D to 3D segmentation [45]. Although more time-consuming, 3D segmentations capture more tumor information. Another advantage of 3D tumor and vessel segmentations is that the segmented CECT scans and 3D reconstructions of the segmentations can help the surgeon prepare for surgery. Furthermore, we performed an interobserver test for the stability of the tumor features to select the more stable features and reduce the number of features. Finally, we extracted handcrafted vessel features to quantify the arterial and venous tumor–vessel contact of all relevant adjacent vessels, while most other radiomics studies only segment the tumor itself or include only one artery or vein.

## 5. Conclusions

In this exploratory study, we evaluated the potential of CT-based radiomics in pancreatic head PDAC to predict resectability in chemo-naïve patients. We trained three SVM classifier models including tumor and/or vessel features that accurately predicted resectability. The performance of the two best performing models was comparable to the performance of the MDT of our tertiary expertise center. Our clinician-independent models show that it is useful to add vessel features to predict resectability. With these encouraging results, more elaborate and automated models could be developed that are radiologist-independent and reduce workload.

## Figures and Tables

**Table 1:** Patient characteristics in the training set versus the test set.

	Training Set (*N* = 86)	Test Set (*N* = 15)	Total (*N* = 101)	*p*-Value
Age in years, mean (SD)	71.0 (8.6)	68.6 (9.6)	70.6 (8.7)	0.34
Sex, *N* (%)				0.1
Male	49 (57%)	5 (33%)	54 (53%)	
Female	37 (43%)	10 (67%)	47 (47%)	
Resectability according to MDT, *N* (%)				0.8
Resectable	25 (29%) ^1^	5 (33%)	30 (30%)	
Borderline	14 (16%) ^1^	3 (20%)	17 (17%)	
Irresectable	47 (54%) ^1^	7 (47%)	54 (53%)	
CT-reported tumor size in mm, mean (SD)	33.4 (15.3)	32.6 (11.2)	33.3 (14.7)	0.84
Time between CT and surgery in days, median (range)	32 (8–49)	25 (19–53)	30 (8–53)	0.27
Surgery *N* (%)	41 (48%)	8 (53%)	49 (49%) ^1^	0.72
*Resected*	*31 (76%)*	*7 (88%) * ^1^	*38 (78%)*	
*Not resected*	*10 (24%)*	*1 (13%) * ^1^	*11 (22%)*	
No surgery	45 (52%)	7 (47%)	52 (52%) ^1^	

SD = standard deviation; MDT = multidisciplinary team. ^1^: percentages do not total 100 because of rounding.

**Table 2:** Included features for the three models.

(1) Tumor Features Model	(2) Vessel Features Model	(3) Combined Features Model
Shape-based—Least Axis Length	Venous Encasement Angle	Venous Encasement Angle
Shape-based—Major Axis Length	Arterial Encasement Angle	Arterial Encasement Angle
First-Order Statistics—10th Percentile	Venous Contact Length	Venous Contact Length
Gray Level Co-occurrence Matrix—Autocorrelation		Gray Level Dependence Matrix—Dependence Entropy
Gray Level Co-occurrence Matrix—Inverse Variance		
Gray Level Dependence matrix—Dependence Entropy		

**Table 3:** Performance of the three models, at the optimal point according to the Youden index, and the MDT to predict resectability on the training set (*N* = 86) and validation with the test set (*N* = 15).

Model	Sensitivity	*p*-Value Sensitivity Model vs. MDT	Specificity	*p*-Value Specificity Model vs. MDT	PPV	NPV	AUC
(1) Tumor features		0.002 *		0.625			0.76 (0.64–0.85)
Training set, *N* = 86	65% (45–81%)		80% (67–90%)		65% (50-77%)	80% (71–87%)	
Test set, *N* = 15	86% (42–100%)		75% (35–97%)		75% (47–91%)	86% (48–97%)	
(2) Vessel features		1.000		0.031 *			0.92 (0.84–0.96)
Training set *N* = 86	97% (83–100%)		88% (47–100%)		67% (56–76%)	98% (85–100%)	
Test set, *N* = 15	100% (59–100%)		73% (59–84%)		88% (53–98%)	100% (−)	
(3) Combined features		1.000		0.063			0.91 (0.84–0.96)
Training set, *N* = 86	94% (79–99%)		75% (61–85%)		67% (57–77%)	95% (84–99%)	
Test set, *N* = 15	100% (59–100%)		88% (47–100%)		88% (53–98%)	100% (−)	
MDT							
Training set, *N* = 86	97% (83–100%)		84% (71–92%)		77% (65–86%)	98% (86–100%)	
Test set, *N* = 15	88% (47–100%)		100% (59–100%)		100% (−)	88% (53–98%)	

Values are displayed as percentages with the 95% confidence interval in brackets. AUC is also displayed with the 95% confidence interval in brackets. Significance between sensitivity and specificity of the three models and the MDT was calculated using the McNemar test. Significant *p*-values are marked with an *. MDT = multidisciplinary team; PPV = positive predictive value; NPV = negative predictive value; AUC = area under the curve.

**Table 4:** Performance of the three models in predicting R status (R0 vs. R1/2). Performance of the training set (N = 31) and the validation with the test set (N = 7).

Model	Sensitivity	Specificity	PPV	NPV	AUC
(1) Tumor features					0.63 (0.42–0.82)
Training set, *N* = 31	100% (78–100%)	38% (15–65%)	60% (51–69%)	100% (−)	
Test set, *N* = 7	80% (28–99%)	0% (0–84%)	67% (56–76%)	−	
(2) Vessel features					0.67 (0.43–0.85)
Training set, *N* = 31	80% (52–96%)	56% (30–80%)	63% (48–76%)	75% (50–90%)	
Test set, *N* = 7	80% (28–99%)	100% (16–100%)	100% (−)	67% (26–92%)	
(3) Combined features					0.73 (0.51–0.89)
Training set *N* = 31	53% (27–79%)	88% (62–98%)	80% (50–94%)	67% (53–78%)	
Test set, *N* = 7	80% (28–99%)	50% (1–99%)	80% (48–94%)	50% (10–90%)	

Values are displayed as percentages with the 95% confidence interval in brackets. The AUC is also displayed with the 95% confidence interval in brackets. Prediction of R status was not performed by the MDT; therefore, these results are not available. PPV = positive predictive value; NPV = negative predictive value; AUC = area under the curve.

## Data Availability

The data presented in this study are available on request from the corresponding author. The data are not publicly available due to privacy and ethics.

## Supplementary Materials

The following supporting information can be downloaded at: https://www.mdpi.com/article/10.3390/diagnostics13203198/s1.
