# Supplementary material for: Computed Tomography-Based Radiomics Using Tumor and Vessel Features to Assess Resectability in Cancer of the Pancreatic Head
Source: Diagnostics (Basel). 2023 Oct 13;13(20):3198. doi: 10.3390/diagnostics13203198 (PMC10606005; doi:10.3390/diagnostics13203198)
Supplement: Supplementary file 1 [file diagnostics-13-03198-s001.zip › Supplementary files_Radiomics PDAC resectability.pdf]

Supplementary files

Complete list of extracted tumour features (N=107; PyRadiomics package version 3.0.1 [1]), including ICC value of interobserver analysis, according to McGraw & Wong [2]

Table S1. Extracted tumour features with ICC values of interobserver analysis

Class	Feature	ICC value
**Shape-based**	Elongation	0.53
	Flatness	0.90
	Least Axis Length	0.92
	Major Axis Length	0.87
	Maximum 2D Diameter Column	0.84
	Maximum 2D Diameter Row	0.50
	Maximum 2D Diameter Slice	0.90
	Maximum 3D Diameter	0.87
	Mesh Volume	0.93
	Minor Axis Length	0.81
	Sphericity	0.72
	Surface Area	0.91
	Surface Volume Ratio	0.78
	Voxel Volume	0.93
**First Order Statistics**	10th Percentile	0.98
	90th Percentile	0.94
	Energy	0.79
	Entropy	0.94
	Interquartile Range	0.97
	Kurtosis	0.74
	Maximum	0.89
	Mean Absolute Deviation	0.95
	Mean	0.97
	Median	0.97
	Minimum	0.94
	Range	0.93
	Robust Mean Absolute Deviation	0.97
	Root Mean Squared	0.97
	Skewness	0.72
	Total Energy	0.79
	Uniformity	0.95
	Variance	0.93
**Gray Level Co-occurrence Matrix**	Autocorrelation	0.94
	Cluster Prominence	0.79
	Cluster Shade	0.74
	Cluster Tendency	0.93

	Contrast	0.99
	Correlation	0.95
	Difference Average	1.00
	Difference Entropy	0.99
	Difference Variance	0.99
	Inverse Difference	1.00
	Inverse Difference Moment	1.00
	Inverse Difference Moment Normalized	0.88
	Inverse Difference Normalized	0.93
	Informational Measure of Correlation 1	0.97
	Informational Measure of Correlation 2	0.95
	Inverse Variance	1.00
	Joint Average	0.90
	Joint Energy	0.97
	Joint Entropy	0.97
	Maximal Correlation Coefficient	0.91
	Maximum Probability	0.98
	Sum Average	0.90
	Sum Entropy	0.94
	Sum Squares	0.95
**Gray Level Dependence matrix**	Dependence Entropy	0.91
	Dependence Non Uniformity	0.92
	Dependence Non Uniformity Normalized	1.00
	Dependence Variance	1.00
	Gray Level Non Uniformity	0.94
	Gray Level Variance	0.93
	High Gray Level Emphasis	0.94
	Large Dependence Emphasis	1.00
	Large Dependence High Gray Level Emphasis	0.91
	Large Dependence Low Gray Level Emphasis	0.47
	Low Gray Level Emphasis	0.31
	Small Dependence Emphasis	0.99
	Small Dependence High Gray Level Emphasis	0.95
	Small Dependence Low Gray Level Emphasis	0.46
**Gray Level Run Length Matrix**	Gray Level Non Uniformity	0.94
	Gray Level Non Uniformity Normalized	0.94
	Gray Level Variance	0.91
	High Gray Level Run Emphasis	0.94
	Long Run Emphasis	1.00
	Long Run High Gray Level Emphasis	0.90
	Long Run Low Gray Level Emphasis	0.45
	Low Gray Level Run Emphasis	0.30
	Run Entropy	0.92
	Run Length Non Uniformity	0.93
	Run Length Non Uniformity Normalized	1.00

	Run Percentage	1.00
	Run Variance	1.00
	Short Run Emphasis	1.00
	Short Run High Gray Level Emphasis	0.94
	Short Run Low Gray Level Emphasis	0.29
**Gray Level Size Zone Matrix**	Gray Level Non Uniformity	0.95
	Gray Level Non Uniformity Normalized	0.71
	Gray Level Variance	0.85
	High Gray Level Zone Emphasis	0.95
	Large Area Emphasis	0.95
	Large Area High Gray Level Emphasis	0.97
	Large Area Low Gray Level Emphasis	0.52
	Low Gray Level Zone Emphasis	0.19
	Size Zone Non Uniformity	0.92
	Size Zone Non Uniformity Normalized	0.92
	Small Area Emphasis	0.88
	Small Area High Gray Level Emphasis	0.94
	Small Area Low Gray Level Emphasis	0.23
	Zone Entropy	0.84
	Zone Percentage	0.96
	Zone Variance	0.95
**Neighbouring Gray Tone Difference Matrix**	Busyness	0.40
	Coarseness	0.73
	Complexity	0.91
	Contrast	0.91
	Strength	0.54

Collinearity assessment of tumour features

Results of collinearity assessment using Spearman correlation coefficient are shown in a separate excel matrix file. The remaining tumour features after removal of collinear features are shown in Table S2.

Table S2. Remaining tumour features

Class	Feature
**Shape-based**	Flatness
	Least Axis Length
	Major Axis Length
	Maximum 2D Diameter Slice
	Surface Volume Ratio
**First Order Statistics**	10th Percentile
	90th Percentile
	Energy
	Entropy
	Maximum
	Minimum
	Range
	Uniformity
**Gray Level Co-occurrence Matrix**	Autocorrelation
	Contrast
	Correlation
	Inverse Difference
	Inverse Difference Moment Normalized
	Informational Measure of Correlation 1
	Inverse Variance
**Gray Level Dependence matrix**	Dependence Entropy
	Large Dependence High Gray Level Emphasis
	Small Dependence Emphasis
	Small Dependence High Gray Level Emphasis
**Gray Level Size Zone Matrix**	Large Area Emphasis
	Large Area High Gray Level Emphasis
	Size Zone Non Uniformity Normalized
	Zone Entropy
**Neighbouring Gray Tone Difference Matrix**	Contrast

Collinearity assessment of vessel features

From the six vessel features, three showed high collinearity: the arterial vessel-tumour contact-length and contact-area were collinear with the arterial angle of encasement; and the venous tumour-vessel contact-area was collinear with the venous tumour-vessel contact-length. The three remaining vessel features after the collinearity analysis were; venous angle of encasement, venous tumour-vessel contact-length, and the arterial angle of encasement. These features also align with clinical practice. The results of the collinearity assessment of the vessel features is shown in Table S3.

Table S3. Collinearity assessment of vessel features

**Veins**			
	Angle	Length	Contact-area
Angle	1	0.85	0.88
Length	-	1	0.95
Contact-area	-	-	1

**Arteries**			
	Angle	Length	Contact-area
Angle	1	0.96	0.97
Length	-	1	0.98
Contact-area	-	-	1

References

1 van Griethuysen JJM, Fedorov A, Parmar C et al (2017) Computational Radiomics System to Decode the Radiographic Phenotype. Cancer Res 77:e104-e107. Doi:10.1158/0008-5472.Can-17-0339

2 McGraw K, Wong SP (1996) Forming Inferences About Some Intraclass Correlation Coefficients. Psychological Methods 1:30-46. Doi:10.1037/1082-989X.1.1.30
